# Artificial Intelligence and Machine Learning in Pediatric Endocrine Tumors: Opportunities, Pitfalls, and a Roadmap for Trustworthy Clinical Translation

**DOI:** 10.3390/biomedicines14010146

**Published:** 2026-01-11

**Authors:** Michaela Kuhlen, Fabio Hellmann, Elisabeth Pfaehler, Elisabeth André, Antje Redlich

**Affiliations:** 1 Department of Pediatrics, Pediatric Hematology/Oncology, Otto-von-Guericke-University, Leipziger Str. 44, D-39120 Magdeburg, Germany; antje.redlich@med.ovgu.de; 2 Paediatrics and Adolescent Medicine, Faculty of Medicine, University of Augsburg, Stenglinstr. 2, D-86156 Augsburg, Germany; 3 Human-Centered Artificial Intelligence, University of Augsburg, Universitaetsstrasse 6a, D-86159 Augsburg, Germany; fabio.hellmann@informatik.uni-augsburg.de (F.H.);; 4 Institute for Neuroscience and Medicine 4 (INM-4), Forschungszentrum Jülich GmbH, Wilhlem-Johnen-Straße, D-52428 Jülich, Germany

**Keywords:** pediatric oncology, endocrine tumors, machine learning, explainability, risk stratification, techquity, radiomics, ethical AI

## Abstract

Artificial intelligence (AI) and machine learning (ML) are reshaping cancer research and care. In pediatric oncology, early evidence—most robust in imaging—suggests value for diagnosis, risk stratification, and assessment of treatment response. Pediatric endocrine tumors are rare and heterogeneous, including intra- and extra-adrenal paraganglioma (PGL), adrenocortical tumors (ACT), differentiated and medullary thyroid carcinoma (DTC/MTC), and gastroenteropancreatic neuroendocrine neoplasms (GEP-NEN). Here, we provide a pediatric-first, entity-structured synthesis of AI/ML applications in endocrine tumors, paired with a methods-for-clinicians primer and a pediatric endocrine tumor guardrails checklist mapped to contemporary reporting/evaluation standards. We also outline a realistic EU-anchored roadmap for translation that leverages existing infrastructures (EXPeRT, ERN PaedCan). We find promising—yet preliminary—signals for early non-remission/recurrence modeling in pediatric DTC and interpretable survival prediction in pediatric ACT. For PGL and GEP-NEN, evidence remains adult-led (biochemical ML screening scores; CT/PET radiomics for metastatic risk or peptide receptor radionuclide therapy response) and serves primarily as methodological scaffolding for pediatrics. Cross-cutting insights include the centrality of calibration and validation hierarchy and the current limits of explainability (radiomics texture semantics; saliency ≠ mechanism). Translation is constrained by small datasets, domain shift across age groups and sites, limited external validation, and evolving regulatory expectations. We close with pragmatic, clinically anchored steps—benchmarks, multi-site pediatric validation, genotype-aware evaluation, and equity monitoring—to accelerate safe, equitable adoption in pediatric endocrine oncology.

## 1. Introduction

Pediatric endocrine tumors are rare and clinically diverse. Treatment choices must balance oncologic control with preservation of endocrine function, normal growth and development, and the prevention of late effects that span a child’s lifetime. This review concentrates on five entities that dominate pediatric endocrine oncology: differentiated thyroid carcinoma (DTC), medullary thyroid carcinoma (MTC), adrenocortical tumors (ACT), intra- (former termed pheochromocytoma) and extra-adrenal paraganglioma (PGL), and gastroenteropancreatic neuroendocrine neoplasms (GEP-NEN).

DTC is the most frequent endocrine malignancy in children and adolescents, with higher malignancy rates in nodules and more frequent nodal/distant spread than in adults, yet very low disease-specific mortality. Decisions aim to tailor surgery, radioactive iodine (RAI), and surveillance to reduce morbidity while safeguarding control [1,2,3].

Pediatric MTC is uncommon and largely multiple endocrine neoplasia type 2 (MEN2)-associated. Timing of thyroidectomy is genotype-driven (*RET* codon risk), with calcitonin and carcinoembryonic antigen used for follow-up [4,5].

ACT are ultra-rare in pediatrics and biologically heterogeneous. Surgery is central, but outcomes vary widely even within stage, motivating refined prognostication [6,7].

Pediatric PGL have a very high heritable fraction and genotype-specific metastatic risk (e.g., *SDHB*). Care depends on safe pre-operative management, resection completeness, and lifelong genotype-tailored surveillance [8,9,10,11].

GEP-NEN are exceptionally rare in children and adolescents. Contemporary care borrows adult pathways centered on somatostatin receptor (SSTR) imaging and peptide receptor radionuclide therapy (PRRT) for advanced disease. Standardized detection, non-invasive grading surrogates, and consistent response assessment remain pressing needs [12,13,14].

### 1.1. A Brief Primer on AI/ML for Clinicians

AI refers to the broader field of creating computational systems capable of performing tasks that typically require human intelligence. ML is a subset of AI focused on developing algorithms and statistical models that enable systems to learn from data.

In pediatric oncology, the most relevant applications are supervised models trained to predict predefined outcomes—recurrence, survival, treatment response, or complication risk—using structured variables (demography, staging, laboratory data), images (ultrasound, CT, MRI, PET), pathology, or genomics.

Tabular learners (regularized regression, gradient-boosted trees, and survival extensions) work well with clinical variables, whereas convolutional networks dominate image analysis. Because predictions inform care, two properties are critical: predicted probabilities should reflect observed frequencies (calibration), and clinicians should be able to see which inputs/which image part drove the estimate and why (explainability) [15,16,17]. Depending on the questions the user may have regarding the model’s outcome, a distinct explanation must be applied. In case the user wants to investigate which input features were completely irrelevant to the outcome, Alterfactual Explanations should be used [18]. To compare the model’s prediction with a healthy/sick version of the input of the same patient, Counterfactual Explanations would create appropriate complementary results [19]. Another method, primarily used for image explanations, is the use of saliency maps, where each pixel in the provided image is highlighted based on the importance leading to the model’s outcome. This approach showed promising results in analyzing adult patients’ pain level based on their facial expressions [20]. Reporting should therefore pair discrimination metrics with calibration curves and provide case-level explanations that are clinically coherent. In pediatrics, prospective evaluation and human oversight are prerequisites before clinical use.

Patient-facing AI—such as chatbots, digital navigators, and symptom-tracking assistants—aims to support children and families with education, logistics, and self-management across the care pathway. Many such tools are powered by large language models (LLMs) for natural-language interaction and, increasingly, by large multimodal models (LMMs) that process both text and images to provide integrated responses. Although this review focuses on clinician-facing decision support, we briefly note patient-facing tools where they intersect with pediatric endocrine oncology.

**Figure 1:**
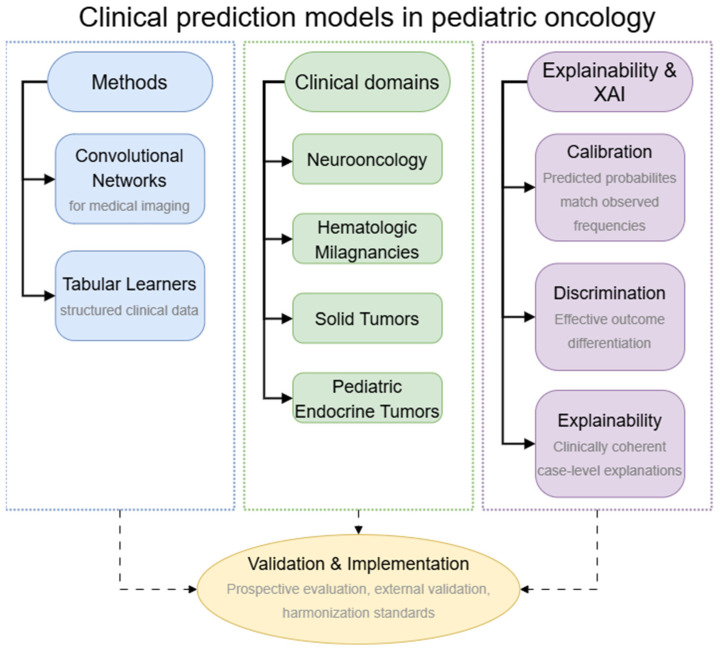
Schematic overview of clinical prediction models in pediatric oncology, distinguishing core method families (blue), key clinical domains (green), and evaluation and explainability pillars (purple) that together support validation and implementation through prospective and external evaluation, harmonization, and human oversight.

Figure 1 summarizes the main method families, pediatric oncology domains, and evaluation and explainablility concepts that structure this review, highlighting how calibration, discrimination, and case-level explanations underpin validation and implementation.

### 1.2. Current Evidence on AI/ML in Pediatric Oncology

AI/ML has matured unevenly across pediatric oncology [21,22,23,24]. Evidence is most advanced in neuro-oncology, where deep learning for detection, segmentation, grading surrogates, and treatment-response assessment appear regularly. The last two tasks are also often addressed by radiomic analysis. Nonetheless, truly independent external validation and clinical-impact studies remain uncommon, and performance can degrade across scanners and sites due to domain shift [25,26,27,28]. Hematologic malignancies have seen steady progress in risk stratification and minimal-residual-disease support tools [29,30,31,32,33,34,35,36]. In solid tumors, retrospective, single-center radiomics and deep learning pipelines are proliferating for diagnosis and survival prediction, yet many lack harmonized acquisition protocols, rigorous calibration, pre-specified action thresholds, and interpretability—all barriers to translation [37,38,39,40]. Multi-institutional efforts to standardize imaging, define pediatric-specific outcomes, and harmonize data are emerging but are not yet routine [41,42].

### 1.3. AI/ML in Pediatric Endocrine Tumors

Against this backdrop, pediatric endocrine tumors present both an opportunity and a stress test: DTC offers large enough cohorts for interpretable recurrence-risk modeling, ACT and PGL demand models that remain reliable at rare-disease scale and across genotypes, and GEP-NEN require method transfer from adult datasets with careful pediatric validation. Progress has been slow because cohorts are small and genetically heterogeneous (e.g., *RET*, *SDHx*, *TP53*), outcomes accrue over long horizons, imaging and assay protocols vary across centers, and cross-border data sharing (general data protection regulation; GDPR) and assent/consent requirements add friction to aggregation.

This review responds with a pediatric-first, entity-structured synthesis of AI/ML applications in DTC, MTC, ACT, PGL, and GEP-NEN, mapped to specific clinical questions (e.g., diagnosis, prognosis, treatment response). We clearly separate pediatric from adult-only evidence (the latter used as methodological context), consolidate studies in a Table 1, and distill methodological and clinical guardrails aligned with contemporary reporting/evaluation standards in Table 2. We also outline an EU-anchored route to harmonized, multi-site validation via the European Cooperative Study Group for Pediatric Rare Tumors (EXPeRT), and the European Reference Network for Paediatric Oncology (ERN PaedCan), with priority on clinically meaningful outcomes rather than algorithmic metrics alone.

## 2. Methods

We reviewed peer-reviewed studies that developed, validated, or evaluated AI/ML tools for diagnosis, prognosis, treatment response, or clinical decision support in children and adolescents with the five endocrine tumor entities of interest: DTC, MTC, ACT, PGL, and GEP-NEN. “Pediatric” was defined as birth through 18 years of age. Mixed-age studies were eligible when pediatric results were reported separately, or a pediatric subgroup could be extracted with reasonable fidelity. Purely adult cohorts were excluded except where (i) the methodology was exemplary and clearly informative for pediatric translation in the same tumor family, or (ii) guidance documents (reporting standards, evaluation frameworks, or regulatory materials) were necessary to contextualize pediatric adoption. We excluded case reports, editorials, letters, conference abstracts without peer-reviewed full texts, and preprints unless later published.

Searches were performed in PubMed/MEDLINE from inception to 30 October 2025 using Boolean combinations of pediatric terms, tumor entity terms, and AI/ML terms (verbatim queries in Appendix A Table A1). We also hand-searched reference lists of included papers and recent reviews and used citation tracking to identify additional records. Contemporary healthcare-AI guidance (e.g., TRIPOD-AI, PROBAST-AI, STARD-AI, SPIRIT-AI/CONSORT-AI, DECIDE-AI, CLAIM, METRICS) and relevant European regulatory materials were consulted to frame evaluation standards [43,44,45,46,47,48,49,50]. Titles/abstracts were screened, followed by full-text review. We extracted study characteristics including population (age range, tumor entity, sample size, setting), data sources (imaging modality, laboratory/clinical variables, and—when available—omics), model class and training scheme, outcomes and time horizons, validation strategy (internal resampling, temporal split, geographic external testing), performance metrics (discrimination and calibration), explainability, and any decision-curve or utility analyses.

Given the expected small number of pediatric endocrine tumor studies and the heterogeneity of designs, inputs, and endpoints, we conducted a narrative synthesis organized by tumor entity and clinical question. For most entity–task pairs, pediatric evidence comprised one or no studies (often k ≤ 1), with non-comparable outcomes, variable imaging/assay protocols, and absence of calibration and action-threshold reporting. Accordingly, no meta-analysis or semi-quantitative pooling was attempted. When comparable pediatric and adult evidence existed, adult-only studies were treated as methodological scaffolding and labeled explicitly to avoid overgeneralization. Emphasis is placed on validation approach, calibration reporting, transparency/interpretability, and reporting completeness.

This review was not prospectively registered. Its scope mirrors the five entities defined in the Introduction. Any updates to the search after 30 October 2025 will be described at submission if applicable.

## 3. AI/ML Applications in Pediatric Endocrine Tumors

Throughout this section, for each entity, studies are discussed in relation to the decision they could inform if validated: (i) triage/detection; (ii) risk estimation for treatment planning and follow-up; (iii) peri-operative safety; and (iv) treatment response. Reported discrimination is noted alongside validation and calibration when available. Clinical use would depend on external pediatric testing and pre-specified action thresholds aligned with existing care pathways (Section 4; Table 2).

### 3.1. Differentiated Thyroid Carcinoma

Ultrasound malignancy triage: A transfer-learned ultrasound system (AI-Thyroid) evaluated in children and adolescents separated malignant from benign nodules with high discrimination and outperformed ACR-TIRADS/K-TIRADS in head-to-head comparisons. The retrospective design and non-standardized image acquisition temper enthusiasm, as do the absent calibration and decision-impact analyses (e.g., avoided biopsies). Explanatory attributions did not consistently map onto familiar sonographic characteristics, limiting bedside interpretability [51].

A second single-center series spanning children and young adults underscored the pediatric trade-off: sensitivity remained high, but specificity was modest compared to radiologists and TI-RADS, highlighting the need for predefined thresholds that account for pediatric tolerance of missed cancers and unnecessary fine-needle aspirations (FNA) [52].

In practice, any ultrasound model would need to present calibrated probabilities tied to FNA vs. observation policies and to perform robustly across vendors and protocols.

Early non-remission/recurrence: In a multi-center pediatric registry (GPOH-MET, *n* = 250), an interpretable gradient-boosted model predicted a 24-month composite of failure to achieve remission or structural recurrence with strong discrimination on an independent test split. Postoperative thyroglobulin, metastatic status at presentation, and very young age consistently shaped estimates in case-level explanations suitable for tumor-board review. Limitations include the retrospective design, Europe-centric cohort, and the absence—so far—of prospective maintenance calibration and decision-curve analyses aligned to pediatric management thresholds [53].

If validated prospectively, such tolls could help individualize the extent of lymph-node dissection, the indication and activities of RAI, and surveillance intensity after initial therapy.

In adult DTC, AI/ML for ultrasound triage and nodal assessment is comparatively mature, and large recurrence/non-remission models increasingly report transparent variable effects and calibration checks. Generalizability across devices/vendors and consistent calibration remain persistent obstacles, so these studies serve mainly as methodological templates rather than evidence for pediatric deployment [54]. AI/ML studies in adult cohorts relevant to pediatric endocrine tumors are detailed in Appendix A Table A2.

Translation hinges on pediatric multi-site testing, assay- and scanner-level calibration (thyroglobulin and ultrasound), and predefined action thresholds linked to FNA, surgery, RAI, and follow-up. Entity-agnostic points on interpretability are summarized in Section 4 and Table 2.

### 3.2. Medullary Thyroid Carcinoma

No pediatric-only ML models for prognosis or surveillance were identified.

Adult cohorts suggest that ultrasound radiomics and combined ultrasound-plus-serology nomograms can stratify nodal risk pre-operatively [55,56], while *RET*-variant triage tools are being used as adjuncts in clinical genetics workflows [57]. Most adult series are retrospective and single- or dual-center with variable calibration reporting.

Direct pediatric use would require genotype-aware models that account for age and calcitonin kinetics, pediatric-tuned ultrasound features, and thresholds linked to MEN2-driven surgical timing and compartment planning—none of which have been shown to date.

### 3.3. Adrenocortical Tumors

Survival prediction (clinical features): A national pediatric registry (GPOH-MET) derived an interpretable survival model from four readily available variables (distant metastasis, tumor volume, pathologic T stage, and resection status). Discrimination was excellent on an internal test set, and individualized survival curves aided communication at the bedside. Explanations revealed non-linear effects, including a data-guided tumor-volume inflection slightly lower than conventional cut-points. The study is limited by single-registry derivation, retrospective data curation, incomplete germline information (e.g., *TP53*), and the abscence of pediatric external validation with update rules [58].

Properly validated, such a parsimonious model fits rare-disease realities and supports equity by avoiding dependence on advanced assays.

Urinary steroid metabolomics (diagnosis/differentiation): A complementary analysis used supervised learning on targeted gas chromatography-mass spectrometry urinary steroid profiles to distinguish ACT from controls and to separate adrenocortical carcinoma (ACC) from adrenocortical adenoma (ACA).

The signal is intriguing but rests on internal validation only, without calibration or decision-impact evaluation, and with the usual concerns about batch and protocol effects in single-laboratory pipelines [59]. As an adjunct, this line of work will need multi-center assay harmonization and external testing before it can be fused with clinical and imaging data in pediatric pathways.

Adult ACC studies provide methodological templates—from clinical-only survival tools to multi-omics prognostics—but their resource demands and cohort structures limit portability to pediatrics without careful adaptation [60,61,62,63,64,65,66,67].

### 3.4. Pheochromocytoma and Paraganglioma

We found no pediatric-only AI/ML models.

Adult studies indicate that biochemical ML scores built from age, pre-test risk, and plasma metanephrines/methoxytyramine can outperform clinicians’ initial estimates. However, simply displaying probabilities to specialists did not meaningfully change final interpretations, underscoring that workflow integration and action thresholds are essential [68]. Clinical-parameter models have predicted intra-operative hemodynamic instability with encouraging accuracy and included calibration and decision-curve analyses; feature attributions emphasized inflammatory and coagulation markers [69]. Imaging signatures derived from venous-phase CT have demonstrated externally validated discrimination for metastatic potential and prognostic value for metastasis-free survival, but they were trained in high *p* ≫ *n* settings with limited reporting on feature stability and calibration, warranting caution [70].

For pediatric translation, genotype-aware evaluation (*SDHB*/*SDHD*/*VHL*), harmonized protocols, and external pediatric testing are prerequisites. Screening or peri-operative models would need thresholds embedded in tumor-board workflows rather than stand-alone probability displays.

### 3.5. Gastroenteropancreatic Neuroendocrine Neoplasms

No pediatric-only AI/ML studies were identified.

In adults, multi-center radiomics across CT and MRI has repeatedly separated lower- from higher-grade disease and estimated nodal status with good discrimination, while lesion-level SSTR-PET radiomics for PRRT response show moderate performance that has not yet translated into patient-level benefit [71,72,73,74].

Any pediatric adoption would require harmonized reconstruction, external pediatric testing, and prospective evaluations that ask whether imaging-based stratification actually changes operative planning or PRRT selection. Equity monitoring is salient where access to SSTR-PET and PRRT is uneven.

### 3.6. Patient-Facing AI—Cross-Entities

We did not identify endocrine-specific, pediatric patient-facing tools. In broader pediatric oncology, small studies of general-purpose chatbots and digital navigators suggest that they can improve accessibility and task completion for basic education and logistics (appointments, fasting instructions, symptom diaries), but clinical accuracy varies and tolls are unsuited to treatment advice or center selection without expert curation and escalation pathways [75].

Adjacent pediatric fields report similar patterns: symptom-triage assistants that escalate fever, pain, or nausea to nurses; pre-operative preparation and survivorship education delivered via reading-level-adaptive chat; and adherence reminders and care-coordination prompts for families who travel long distances. These prototypes typically demonstrate usability gains and knowledge recall rather than patient-level outcome changes, reinforcing the need for limited scopes, plain-language outputs, and explicit handoffs to clinicians [76,77,78,79,80,81,82].

Ongoing reliability efforts: To reduce error and drift, current pilots increasingly (i) ground chatbot answers in curated, locally approved pediatric content (guidelines, patient leaflets) via retrieval-augmented generation; (ii) use safety classifiers/abstention rules to block dosing or treatment recommendations and trigger escalation; (iii) implement structured intent detection (education, logistics, symptom check) with role-appropriate responses; (iv) log interactions for quality review and subgroup monitoring (language, age band); and (v) provide offline/low-bandwidth modes to support equitable access.

For pediatric endocrine translation, a scoped navigator for *MEN2*/*PGL*/*DTC* could handle scheduling, test preparation (e.g., biochemical sampling requirements), and travel letters, while abstaining from advice on dose changes or surgical timing and routing such questions to the MDT.

In adult thyroid and endocrine cancers, evaluations of LLM-based chatbots provide readable answers to common questions but show variable accuracy on management topics, frequent omissions, and no evidence of calibration or patient-level benefit. Most studies are cross-sectional and platform-specific [83,84,85].

### 3.7. Multi-Omics, Gene Expression, and Network-Based AI—Cross-Entities

No pediatric endocrine tumor studies developing or externally validating gene expression, network-based, or multi-omics ML models were found.

Adult work illustrates how expression signatures, co-expression networks, radiogenomic links, and multi-omics fusion can support subtyping, risk stratification, and nodal prediction in related endocrine tumors [86,87,88,89,90,91].

These pipelines are best viewed as methodological scaffolding that would require pediatric biospecimen harmonization, explicit batch-effect control, conservative feature spaces relative to sample size, and pediatric external validation with calibration.

A summary of the identified studies on AI/ML applications in pediatric endocrine tumors is provided in Table 1.

**Table 1:** AI/ML studies in pediatric endocrine tumors.

Ref./Entity	Data Modality	Task/Endpoint	Algorithms	Validation	Performance	Limitations
Ha et al. 2025 [51]/Thyroid nodules, two sites, *n* = 128	Ultrasound	Benign vs. malignant nodule classification	DL model (AI-Thyroid, transfer-learned from adult data)	Two pediatric cohorts; plane-specific testing	AUROC 0.913–0.929; sensitivity 79–89%; specificity 80–92%	Pilot; retrospective; external pediatric-only training not used
Yang et al. 2023 [52]/Thyroid nodules (children and young adults), single-center, *n* = 139	Ultrasound	Compare radiologists, ACR TI-RADS, and DL algorithm	CNN-based classifier	Internal test set	Sensitivity 87.5%; specificity 36.1% (DL model)	Mixed age band; needs external validation
Redlich et al. 2025 [53]/DTC, national registry *n* = 250	Routine clinical + biochemical, metastasis status	Predict non-remission/recurrence within 24 months	Gradient-boosted trees (XGB) with SHAP	Stratified hold-out test set with 50 bootstrap resamples	AUROC ≈ 0.86 (test); mean ≈0.82 across resamples	Retrospective; needs prospective and external validation
Redlich et al. 2025 [58]/ACT, national registry, *n* = 97	Routine clinical variables	Individualized survival prediction	XGB-Cox with SHAP	Stratified train/test; 500-bootstrap estimation	C-index 0.925 (test); bootstrap mean 0.891; IBS ≈ 0.09	Retrospective; single-registry
Wudy et al. 2025 [59]/ACT, national registry, *n* = 46	Urinary steroid GC–MS metabolomics	Tumor detection (ACT vs. controls) and ACC vs. ACA differentiation	Logistic regression; decision tree; PCA/clustering (exploration)	Internal only	Not provided	Multi-center external validation needed

Abbreviations: ACA, adrenocortical adenoma; ACC, adrenocortical carcinoma; AU(RO)C, area under the (receiver operating characteristic) curve; C-index, concordance index; CNN, convolutional neural network; CV, cross-validation; DL, deep learning; DTC, differentiated thyroid carcinoma; GEP-NEN, gastroenteropancreatic neuroendocrine neoplasm; LNM, lymph node metastases; MLP, multilayer perceptron; PGL, paraganglioma; PanNET, pancreatic neuroendocrine tumors; PRRT, peptide receptor radionuclide therapy; RF, random forest; SHAP, SHapley additive explanations; SVM, support vector machine; US, ultrasound; XGB, XGBoost.

## 4. Methodological Guardrails for Pediatrics

Checklists help readers know what to report; they do not tell pediatric teams how to build reliable tools in rare, genotype-diverse diseases. This section distills practical guardrails for pediatric ETs and indicates where common AI reporting frameworks fit—and where they do not.

Problem formulation and endpoints: Pediatric ET decisions cluster around four domains: triage/detection (e.g., thyroid FNA vs. observation), risk estimation for treatment planning and follow-up (e.g., early non-remission in DTC; survival in pACT), peri-operative safety (e.g., PPGL hemodynamic instability), and treatment response (e.g., imaging surrogates for grade/PRRT response). Credible studies define actionable endpoints and time-at-risk windows up front, specify inclusion/exclusion with temporality (to avoid leakage), and state thresholds aligned to existing pathways. Ambiguous composites (e.g., lesion-level signals used to infer patient-level benefit) should be avoided or clearly justified. TRIPOD-AI supports clarity for prediction models [43] and STARD-AI helps diagnostic accuracy [45], but neither dictates pediatric thresholds—these must be pre-specified with clinician input.

Data provenance, labeling, and harmonization: Label quality and site effects drive most downstream failures. For imaging, acquisition and reconstruction must be documented (ultrasound presets; CT kernel/slice thickness; MRI sequence; PET reconstruction). When radiomics is used, Image Biomarker Standardization Initiative (IBSI; [92])-conformant feature definitions and reporting of resampling/quantization are essential, and feature stability (test–retest, inter-scanner, or phantom) should be shown. For clinical/biochemical variables, pediatric reference ranges and assay variability (e.g., thyroglobulin, calcitonin) should be explicit; for omics, batch correction and cross-platform normalization are required. Harmonization methods (e.g., ComBat; [93]) must be specified. CLAIM 2024 and METRICS (radiomics) cover much of this [49,50], but ultrasound specifics and pediatric ranges often need additional local detail.

Clinician’s note: For radiomics, use IBSI-conformant features and show they are stable across scanners. For labs like thyroglobulin/calcitonin, name the assay and range so risks can be compared across sites.

Small-N analysis and uncertainty: Pediatric ET cohorts are small and genetically heterogeneous (*RET*, *SDHx*, *TP53*). Analyses should acknowledge the *p* ≫ *n* regime: constrain feature spaces, prefer parsimonious or regularized models when performance permits, use learning-curve plots, and avoid optimistic single splits. Internal validation should use bootstrap or nested cross-validation with leakage safeguards. Where feasible, add temporal and geographically external tests. TRIPOD-AI and PROBAST-AI help structure these choices but do not replace transparent code/configuration sharing [43,46].

Clinician’s note: In small cohorts, prefer models that keep features few and stable. A slightly lower AUROC with good calibration and transparent variables usually outperforms a complex model when you move between centers.

Calibration, thresholds, and clinical utility: Discrimination alone does not determine use. Pediatric studies should report calibration (plot/metrics; calibration-in-the-large and slope), define action thresholds linked to concrete actions (FNA, extent of surgery/RAI, alpha-blockade plan, surveillance cadence), and include decision-curve analysis using those thresholds [15,94,95]. External pediatric validation should confirm both calibration and net benefit. DECIDE-AI encourages early clinical evaluation but does not prescribe thresholds; pediatric teams must do so [48].

Clinician’s note: In practice, a “10% risk” should correspond to ~10 out of 100 similar patients actually experiencing the event over the defined time horizon. When that is not true, recalibration (slope/intercept adjustment) is required before you set action thresholds.

Interpretability and human oversight: In pediatrics, explanations support verification and communication, not mechanism proof. SHAP attributes which inputs most influenced a prediction but does not ensure those inputs map to meaningful clinical constructs or generalize them. Saliency/attention maps show where a model looked, not which properties were decisive. Many radiomic textures lack stable, biologically intuitive semantics and can vary with acquisition. Practical mitigations include IBSI-conformant feature spaces, stability checks, constraining features when possible, and pre-specifying the clinical concepts explanations should reflect. Evaluation should include usefulness/appropriate-reliance endpoints (decision quality, threshold adherence, time to decision, cognitive workload) distinct from model trustworthiness (calibration/robustness/bias). CLAIM and DECIDE-AI support reporting, but clinician-co-designed interfaces are often the missing ingredient [48,49].

Clinician’s note: Treat explanations as verification aids; ask whether the top features or image regions align with recognizable clinical constructs (e.g., margins, echogenicity, microcalcifications in DTC)—and if not, pause, because attribution ≠ mechanism.

Subgroups, fairness, and safety: Performance and calibration should be reported by age bands, sex, ancestry, genotype (e.g., *SDHB*, *TP53*), and site/vendor strata. Failure-mode analyses, missing-data patterns, and safeguards against data leakage are needed. For patient-facing tools, scope should remain education/navigation with explicit escalation to clinicians. Subgroup comprehension and accessibility merit monitoring. Standards mention subgroup reporting but rarely define pediatric-relevant strata—teams must choose them a priori [43,49,96].

Regulatory posture and lifecycle: If clinical deployment is envisioned, documentation should anticipate European Union (EU) AI Act (high-risk) expectations, Food and Drug Administration (FDA)/International Medical Device Regulators Forum (IMDRF) Good ML Practice, and national device rules. That includes change-control plans (pre-determined update procedures), drift surveillance, recalibration triggers, rollback procedures, and audit trails. In Europe, leveraging ERN PaedCan/EXPeRT governance can streamline oversight and cross-border collaboration. SPIRIT-AI/CONSORT-AI cover trial reporting, but pediatric ETs often require pragmatic designs (cluster/stepped-wedge) rather than classic RCTs [44].

Where the standards apply—and where they fall short:

TRIPOD-AI/PROBAST-AI: Strong for what to report and risk-of-bias appraisal in prediction models. They do not specify pediatric action thresholds or genotype-aware subgroup sets.

CLAIM 2024: Imaging reporting is comprehensive. Pediatric ultrasound variance and center-specific presets often demand extra local detail.

STARD-AI: Useful for diagnostic accuracy, but lesion-level tasks and segmentation outputs require careful mapping to patient-level decisions.

METRICS (radiomics)/*IBSI*: Define reporting and features but not biological meaning or stability requirements—pediatric ETs should add test–retest/inter-scanner checks.

DECIDE-AI: Orients early clinical evaluation. It does not replace pre-specification of pediatric thresholds or utility endpoints.

SPIRIT-AI/CONSORT-AI: Trial protocols/reporting are well defined. Feasibility in very rare pediatric ETs often points to stepped-wedge/cluster designs and registry-based endpoints.

A pediatric ET–focused, at-a-glance checklist that operationalizes these points appears in Table 2.

Practical examples of these methodological guardrails (e.g., calibration and decision curves) are provided in Appendix A Box A1, Box A2, Box A3 and Box A4.

**Table 2:** Methodological and clinical guardrails for AI/ML in pediatric endocrine tumors, mapped to reporting/evaluation standards.

Guardrail Topic	What It Means	Pediatric ET-Specific Application	Checklist Anchors
Problem specification, outcomes	Clearly define intended use and actionable endpoints/time-at-risk windows	e.g., DTC: 24-month non-remission/recurrence; pACT: disease-specific/overall survival horizons; PGL: intra-op instability risk; GEP-NEN: PRRT response endpoints	TRIPOD-AI, STARD-AI (diagnostic tasks), SPIRIT-AI (protocols)
Cohort construction, risk of bias	Transparent inclusion/exclusion, temporality, leakage safeguards; appraisal of bias	Exclude post-outcome variables; align imaging/biochemistry windows; report flow diagrams	TRIPOD-AI; PROBAST-AI (risk-of-bias appraisal); CLAIM
Data governance, consent	Describe consent/assent, de-identification, data use agreements, minimization of unnecessary elements	pediatric assent; family privacy; data use restrictions	CLAIM (data), SPIRIT-AI/CONSORT-AI (ethics), institutional/GDPR notes
Reference standards	Define ground truth and adjudication; report reader agreement	e.g., Thyroid nodule histology; PGL risk by Grading of Adrenal Pheochromocytoma and Paraganglioma; PRRT response definitions; centralized pathology	STARD-AI, CLAIM, TRIPOD-AI
Preprocessing, harmonization	Missing-data strategy; image normalization; batch/site correction; radiomics standards	Cross-vendor US/CT/MRI; PET recon settings; assay variability	CLAIM; METRICS (radiomics); IBSI conformance
Sample size, analysis plan	Justify size; prespecify analysis/stop rules; plan for small-N uncertainty	Rare pACT/PGL: multi-registry pooling; federated learning; learning-curve plots	TRIPOD-AI; PROBAST-AI (appraisal); DECIDE-AI (pilot evaluation)
Modeling transparency	Report algorithms, hyperparameters, versioning, and rationale	Document transfer-learning for pediatric US; share configs/code where possible	TRIPOD-AI, METRICS, CLAIM
Validation (internal, external)	Use bootstrap/nested CV; temporal split; independent multi-site tests	Train in registry A, test in registry B; temporal split around guideline changes	TRIPOD-AI; STARD-AI; DECIDE-AI (early clinical studies)
Calibration, clinical utility	Provide calibration plots/metrics; decision-curve analysis with clinical thresholds	e.g., DTC: biopsy vs. observe; pACT: adjuvant discussion; PGL: alpha-blockade intensity	TRIPOD-AI; CLAIM; DECIDE-AI; CONSORT-AI (impact)
Subgroups, fairness, safety	Prespecify subgroup analyses; report performance and calibration by subgroup; failure modes	Age bands, sex, ancestry; genotype (*SDHB*, *VHL*, *TP53*); scanner/vendor strata	TRIPOD-AI; CLAIM; SPIRIT-AI/ CONSORT-AI (safety reporting)
Explainability, human-in-the-loop	Provide case-level explanations; describe clinician oversight and review points	SHAP for tabular models; heatmaps for US; pre-specified clinical concepts	TRIPOD-AI; CLAIM; DECIDE-AI (human factors)
Deployment description	Specify electronical medical record/radiology information system integration, alerting, user roles, and escalation	MDT dashboards; embargo on auto-finalization; CPMS tumor-board context	SPIRIT-AI/ CONSORT-AI; DECIDE-AI
Monitoring, updates	Drift checks, recalibration, change-control plans, rollback procedures	Annual re-validation; pediatric threshold review post-guideline updates	TRIPOD-AI; CONSORT-AI; DECIDE-AI
Data, code availability	Share de-identified/synthetic data where possible; reproducible code and model cards	Synthetic pediatric US; model cards with pediatric performance notes	TRIPOD-AI; METRICS; CLAIM
Multi-omics integration and network methods	Define fusion strategy (early, intermediate, late), batch correction, and causal/graph assumptions, document assay quality control and feature stability	e.g., DTC/MTC: integrate genotype with imaging/biochemistry; pACT: combine clinical data with urinary steroidomics; PGL: genotype-aware biochemical and imaging fusion	TRIPOD-AI; DECIDE-AI; METRICS

## 5. Ethics, Equity, and Patient-Facing AI

Why pediatrics is different: Children’s longer life expectancy, evolving physiology, and dependence on guardians make risk–benefit trade-offs are fundamentally different from those in adult oncology. Recent pediatric ethics statements and viewpoints from the American Academy of Pediatrics emphasizes pediatric-specific governance, proportionate oversight, and the enrichment of pediatric data resources through collaboration and responsible sharing—for example, harmonized registries, age-appropriate consent/assent, and privacy-preserving analytics—so that AI systems are both safe and representative [96]. In this framing, the goal is not to collect less data per se but to collect the right data under robust safeguards, minimizing unnecessary elements while maximizing quality, inclusiveness, and long-term stewardship.

Patient-facing AI/LLMs—promise and pitfalls: Evaluations in pediatric oncology show that general-purpose chatbots can provide accessible information but are not adequate for treatment guidance, center selection, or nuanced counseling without expert curation and clear escalation pathways [75]. Reliability and safety can be operationalized with grounded content (answers limited to locally curated pediatric materials), hard stops for out-of-scope queries (dose changes, urgent symptoms) with automatic escalation, and appropriate-reliance metrics (comprehension checks, escalation rates, resolution times) tracked by subgroup to detect inequities. Institutions can require vendor model cards, update logs, and incident reporting and embed tools within ERN PaedCan/EXPeRT governance so consent/assent, privacy, and accessibility reviews are standardized rather than ad hoc.

Techquity—the equity lens: Digital innovations can widen disparities if poorly designed, but—as argued in recent work on pediatric and adolescents and young adult oncology—generative AI and immersive technologies can also actively reduce inequities when built and governed for techquity. Examples include multilingual, reading-level-adaptive counseling materials, culturally contextualized education co-created with families, low-bandwidth and offline delivery options, and standardized, immersive procedural preparation that narrows variation in pre-treatment information. In this framing, the actionable levers are design and measurement (co-design with under-served groups, subgroup performance and comprehension audits, continuous content localization), rather than generic calls to “ensure access”, with governance focused on documenting gaps and closing them iteratively [97].

Global and institutional governance: The World Health Organization’s 2024–2025 guidance on LMMs in health frames and end-to-end, risk-managed lifecycle includes pre-deployment evaluation for clinical accuracy and harms, content provenance/labeling of AI-generated outputs, mandatory human oversight, and transparent documentation of training data sources and model limitations. It further emphasizes privacy-by-design and data minimization, bias and accessibility audits (with attention to children and guardians), and post-deployment surveillance with incident reporting and governed updates. Procurement clauses (e.g., model cards, update logs, data-use restrictions, cybersecurity) are recommended to operationalize these expectations. (ISBN: 978-92-4-008475-9).

Field-applicable recommendations with illustrative cases: Translating principles into practice in pediatric ETs often comes down to scoping, governance, and measurement. Three brief scenarios illustrate how the ethics/equity guardrails can be operationalized without overpromising what AI can do.

Case A—DTC ultrasound triage: narrow scope, calibrated thresholds, and human review. In a pilot where an ultrasound model flags higher-risk thyroid nodules, outputs are limited to calibrated probabilities mapped to a pre-agreed FNA threshold set by the MDT for the local pediatric population. The model never auto-orders biopsies. Instead, a short note explains which recognizable sonographic traits (e.g., margins, echogenicity, microcalcifications) were most influential, and the radiologist/endocrinologist retains decision authority. Equity is monitored through routine reports stratified by age band, sex, and site/vendor, and a simple appeal path (MDT re-read) is available for families. This design aligns patient safety (no automation), transparency (thresholds published), and appropriate reliance (MDT sign-off).

Case B—PPGL peri-operative instability: safety-first integration. Where a clinical-parameter model estimates the risk of intra-operative hemodynamic instability, its output triggers a pre-anesthesia huddle and prompts documentation of the alpha-blockade plan. No changes occur automatically. The team agrees in advance on actions at low/indeterminate/high risk, and adverse events are tracked prospectively as part of a post-deployment safety log. To minimize bias, performance is periodically reviewed by genotype (e.g., *SDHB*/*SDHD*/*VHL*), and thresholds are re-evaluated after guideline changes. This approach embeds human oversight, genotype-aware equity, and lifecycle monitoring.

Case C—Patient-facing navigator for MEN2 families: educate, don not advise. A lightweight, multilingual navigator is scoped to education and logistics (appointments, travel letters, pre-op fasting rules) and avoids treatment advice. Content is curated from pediatric guidelines, written at adjustable reading levels, and available offline for bandwidth-constrained settings. The tool recognizes “out-of-scope” questions (e.g., whether to delay surgery) and escalates to the clinical team. Programs evaluate comprehension and trust with brief checks, track errors/omissions, and compare performance across language groups to prevent widening disparities. This keeps patient-facing AI useful while respecting limits.

Across these use cases, procurement can make expectations concrete: model cards (training data domains, pediatric performance, known limits), update logs, data use and retention terms, accessibility requirements (language, reading level, offline mode), and incident reporting pathways. Institutions can house these within existing ERN PaedCan/EXPeRT governance so that pediatric-specific assent/consent, privacy, and equity reviews are routine rather than ad hoc.

## 6. Roadmap for Clinical Translation in Pediatric Endocrine Tumors

Clinical translation in rare, genotype-diverse pediatric endocrine tumors will be incremental and most feasible within existing European infrastructures (EXPeRT, ERN PaedCan, Clinical Patient Management System virtual tumor boards (CPMS), PARTNER) [98,99,100,101]. Below, we outline foundations, pilot designs, and evaluation strategies using concrete, entity-specific examples. The aim is to move beyond “AI potential” toward deployable, auditable decision support.

Foundations: Rather than building pipelines from scratch, centers can map local data to minimal common elements compatible with PARTNER/EXPeRT (core clinical variables; imaging descriptors—contrast phase, slice thickness, reconstruction kernel; biochemistry with assay identifiers; genotype/variant class where available). For radiomics, report IBSI-conformant feature definitions and stability checks (test–retest or inter-scanner). For tabular data, adopt pediatric reference ranges and units. Consent/assent text should anticipate cross-border sharing under GDPR and explicitly cover model evaluation. Within ERN PaedCan, CPMS tumor boards provide a natural venue to surface model cards (intended use, training domains, pediatric performance, limits) without automating orders or reports.

Pragmatic pilots: Pilots should target narrow questions with clear actions, use pre-specified thresholds, and log decisions and rationales.

DTC early non-remission—treatment de-escalation/escalation: Integrate a 24-month non-remission predictor into post-operative MDT review. Map probabilities to explicit actions (e.g., observation vs. compartment dissection vs. RAI activity band) agreed ex ante. Require a short, structured note (model estimate; top contributing factors; clinical decision) to create an audit trail.

ACT survival—risk stratification: Deploy a parsimonious four-variable survival calculator (distant metastasis, tumor volume, pT stage, resection status) as a read-only decision aid during CPMS boards. Before go-live, set a risk threshold (e.g., predicted 3-year disease-specific survival below a pre-agreed cut-point) that triggers discussion of adjuvant therapy or intensified follow-up. Monitor calibration quarterly and record whether the calculator changed the conversation (yes/no; how).

Rationale: aligns with equity (routine variables), suits small-N settings, and provides an immediately interpretable output.

PGL peri-operative instability—safety planning: Use a clinical-parameter model to stratify hemodynamic instability risk at pre-anesthesia conference. Actions are templated (alpha-blockade targets, invasive monitoring, anesthesia staffing). No automation occurs. The model simply prompts a checklist and records adherence.

GEP-NEN imaging—federated radiomics: For adolescent SSTR-PET or contrast CT/MRI tasks (grading surrogate or nodal risk), run federated/distributed training across participating sites coordinated by EXPeRT, avoiding centralization of identifiable images. Harmonize reconstruction settings up front and log site-wise performance to identify drift or bias.

Evaluation and sustainability: When signal and feasibility are shown, scale within networks using quasi-experimental designs (stepped-wedge or cluster roll-out across centers). Outcomes should be clinically anchored and entity-specific: time-to-diagnosis, reduction in avoidable FNAs (DTC) or peri-operative complications (PGL), timeliness of genetics referral, or alignment with MEN2 surgical timing rather than AUROC alone. Each deployment should include the following: (i) calibration maintenance (plots, calibration-in-the-large, slope) at planned intervals; (ii) equity dashboards (performance and calibration by age, sex, ancestry, genotype, site/vendor); (iii) drift surveillance with triggers for recalibration or rollback; and (iv) governed change-control (versioned releases, update logs, incident reporting) aligned to EU AI Act “high-risk” expectations and national device rules. Within ERN PaedCan/EXPeRT, steering groups can serve as oversight bodies for approvals and equity monitoring.

To accelerate replication, each exemplar can ship with a short pack: (1) one-page model card; (2) minimum data dictionary (fields, units, ranges); (3) threshold rationale; (4) calibration-check template; (5) CPMS note template; and (6) monitoring checklist (equity slices; drift flags). These lightweight artifacts make pilots reproducible across centers with different resources.

## 7. Conclusions and Future Directions

AI/ML holds credible promise across pediatric endocrine tumors, provided models are developed on harmonized data, are calibrated and interpretable, and are evaluated prospectively within pediatric care pathways. Progress should prioritize multi-center collaboration, federated analyses, and pragmatic prospective studies with predefined actions and clinically meaningful endpoints, coupled to equity audits and lifecycle monitoring. With this disciplined approach, collaborative networks can translate retrospective signals into safe, reproducible, and equitable clinical benefit for children and adolescents.

## Data Availability

Not applicable.

## Abbreviations

The following abbreviations are used in this manuscript:

ACA	Adrenocortical adenoma
ACC	Adrenocortical carcinoma
ACT	Adrenocortical tumor
AI	Artificial intelligence
AUROC	Area under the (receiver operating characteristic) curve
DTC	Differentiated thyroid carcinoma
ERN PaedCan	European Reference Network for Paediatric Oncology
EXPeRT	European Cooperative Study Group for Pediatric Rare Tumors
FNA	Fine-needle aspiration
GEP-NEN	Gastroenteropancreatic neuroendocrine neoplasm
IBSI	Image Biomarker Standardization Initiative
LLM	Large language models
LMM	Large multimodal models
MDT	Multidisciplinary tumor board
ML	Machine learning
MTC	Medullary thyroid carcinoma
PGL	Intra- and extra-adrenal paraganglioma
PRRT	Peptide receptor radionuclide therapy
SHAP	Shapely Additive exPlanations
XAI	Explainable artificial intelligence

## Appendix A

**Table A1:** PubMed/MEDLINE search strategy.

Search Block	Verbatim Boolean Query (PubMed Syntax)
All endocrine entities (master query)	(“pediatric” OR child * OR adolescen *) AND ((“differentiated thyroid carcinoma” OR “papillary thyroid carcinoma” OR “follicular thyroid carcinoma” OR DTC OR PTC OR FTC OR “medullary thyroid carcinoma” OR MTC OR adrenocortical OR “adrenal cortical” OR “adrenocortical carcinoma” OR ACC OR “adrenocortical tumor *” OR pheochromocytoma OR paraganglioma OR PPGL OR (neuroendocrine AND (gastroenteropancreatic OR pancreatic OR PanNET OR “pancreatic NET” OR “pancreatic neuroendocrine” OR “small intestinal” OR “small bowel” OR midgutOR GEP))) AND (“artificial intelligence” OR “machine learning” OR “deep learning” OR radiomics OR radiogenomics OR “multi-omics” OR omics OR “neural network *” OR “support vector” OR “random forest” OR “gradient boosting” OR XGBoost OR “risk model *” OR “prediction model *” OR “survival model *”)
DTC-focused	(“pediatric” OR child * OR adolescen *) AND (thyroid AND (“differentiated thyroid carcinoma” OR “papillary thyroid carcinoma” OR “follicular thyroid carcinoma” OR DTC OR PTC OR FTC)) AND (“artificial intelligence” OR “machine learning” OR “deep learning” OR radiomics OR “risk model *” OR “prediction model *”)
MTC-focused	(“pediatric” OR child* OR adolescen *) AND (“medullary thyroid carcinoma” OR MTC) AND (“artificial intelligence” OR “machine learning” OR radiomics)
ACT-focused	(“pediatric” OR child * OR adolescen *) AND (adrenocortical OR “adrenal cortical” OR ACC OR “adrenocortical carcinoma” OR “adrenocortical tumor *”) AND (“artificial intelligence” OR “machine learning” OR radiomics OR “risk model *” OR “survival model *”)
PGL-focused	(“pediatric” OR child * OR adolescen *) AND (pheochromocytoma OR paraganglioma OR PPGL) AND (“artificial intelligence” OR “machine learning” OR radiomics)
GEP-NEN-focused	(“pediatric” OR child * OR adolescen *) AND (neuroendocrine AND (gastroenteropancreatic OR pancreatic OR PanNET OR “pancreatic NET” OR “pancreatic neuroendocrine” OR “small intestinal” OR “small bowel” OR midgut OR GEP)) AND (“artificial intelligence” OR “machine learning” OR radiomics OR radiogenomics OR “multi-omics”)

**Table A2:** AI/ML studies in adult cohorts with pediatric relevance.

Ref./Entity	Data Modality	Task/Endpoint	Algorithms	Validation	Performance	Limitations
Pamporaki et al. 2025 [68]/PGL, multi sites, *n* = 2046	Biochemical screening + age + pre-test risk	Screening/diagnostic support: disease-probability score for PGL	ML classifiers (logistic regression/tree-based)	External, multi-site validation; comparison of specialists’ pre- vs. post-score interpretations	ML scores outperformed specialists’ pre-test estimates; negligible change in specialists’ final interpretations	Thresholds not pre-specified; assay standardization required; minimal demonstrated clinical impact; pediatric validation needed
Zhao et al. 2025 [69]/PGL, single center, *n* = 197	Clinical variables, imaging features	Predict intra-op hemodynamic instability	RF, SVM, LightGBM, MLP ensembles	Internal train/test; calibration and decision-curve analyses	Best AUROC ≈ 0.86; good calibration	Etiology and physiology differ in children
Zhou et al. 2025 [70]/PGL, three sites, *n* = 249	CT venous-phase radiomics, DL (ResNet), clinical	Pre-op metastatic potential/high-risk (GAPP ≥ 3)	Six ML models + ResNet features; combined model	External validation across two test cohorts	AUCs > 0.87 across datasets; prognostic for MFS	Requires pediatric imaging harmonization
Gu et al. 2023 [71]/PanNET, two sites, *n* = 320	Contrast-enhanced CT or MRI	Predict grade (G1–G3), LNM, or aggressiveness	DL signatures + radiomics; nomograms	External validation (multi-center)	Typical AUCs 0.85–0.93 depending on task	Prospective pediatric validation lacking
Laudicella et al. 2022 [74]/GEP-NEN, single center, *n* = 38	[68Ga]DOTATOC PET/CT radiomics ± clinical	Predict response to PRRT at lesion level	Feature selection + logistic/discriminant analysis	k-fold CV; per-site analyses	AUC ~0.74–0.75 for histogram skewness; SUVmax non-predictive	Heterogeneous scanners; lesion-level outcomes

Box A1 Calibration in practice (pediatric DTC example). Calibration links predicted risk to what actually happens. Suppose a model estimates a 24-month non-remission risk of 30% for a child after DTC surgery. In a calibrated model, among 100 similar children, about 30 will not remit by 24 months. If, on testing, the observed rate is 18%, the model is over-predicting and should not guide decisions until recalibrated (e.g., adjust intercept/slope). Report a calibration plot and simple statistics (calibration-in-the-large, slope). Decisions (e.g., extent of lymph-node dis-section, RAI activity) should then be tied to pre-agreed thresholds (e.g., intervene if risk ≥ X%) set by the MDT.

Box A2 Decision curves and thresholds (pediatric PGL example). Decision-curve analysis (DCA) checks whether using a model is better than “treat/test all” or “treat/test none” at clinically relevant thresholds. In PGL biochemical screening, a threshold of, say, 10% might reflect the point at which you proceed to additional imaging. Plotting net benefit across thresholds shows whether the model adds value where you actually plan to act. Report the thresholds before you run DCA and interpret the curve where the team will use it—not across the entire 0–100% range.

Box A3 Validation hierarchy.

- Internal validation (bootstrap/nested CV): guards against overfitting in the same data distribution.
- Temporal split: tests resilience to changes over time (protocols, practice).
- Geographically external testing: challenges the model with different scanners, assays, and case-mix—often where pediatric tools fail.
- Prospective evaluation/early deployment: checks usability, calibration maintenance, and safety signals.
- Impact evaluation (cluster/stepped-wedge): asks whether decisions and outcomes actually improve (e.g., fewer avoidable FNAs; fewer peri-operative complications).

Box A4 Reading explanations responsibly (SHAP, saliency, radiomics). Use SHAP to verify that influential tabular features match clinical expectations. Be cautious when high-order radiomic textures dominate without stability evidence. For images, a saliency map that lights up a suspicious margin is reassuring; one that highlights unrelated regions warrants review. Document when explanations changed a decision (or prevented an over-reliant one).
